# Acetazolamide-Loaded Nanoparticle Based on Modified Hyaluronic Acid as Delivery System to Target Carbonic Anhydrases in *Escherichia coli*

**DOI:** 10.3390/ijms26104908

**Published:** 2025-05-20

**Authors:** Valentina Verdoliva, Viviana De Luca, Claudiu T. Supuran, Stefania De Luca, Clemente Capasso

**Affiliations:** 1 Department of Environmental, Biological and Pharmaceutical Sciences and Technologies, Institute of Crystallography, National Research Council (CNR), Via Vivaldi, 43, 81100 Caserta, Italy; valentina.verdoliva@cnr.it; 2 Department of Biology, Agriculture and Food Sciences, National Research Council (CNR), Institute of Biosciences and Bioresources, Via P. Castellino, 111, 80131 Naples, Italy; viviana.deluca@ibbr.cnr.it; 3 Neurofarba Department, Section of Pharmaceutical Sciences, University of Florence, Via Ugo Schiff 6, Sesto Fiorentino, 50019 Florence, Italy; claudiu.supuran@unifi.it; 4 Department of Biomedical Sciences, Institute of Biostructures and Bioimaging, National Research Council (CNR), Via P. Castellino, 111, 80131 Naples, Italy

**Keywords:** acetazolamide, carbonic anhydrase, hyaluronic acid, biomaterial-based drug carrier, bacterial metabolism

## Abstract

Acetazolamide (AZA) is a validated carbonic anhydrase inhibitor (CAI) that has the potential for use in various therapeutic applications. Herein, we report a novel AZA-loaded biodegradable nanodelivery system that was proven to enhance the antibacterial efficacy of the drug against Gram-negative bacteria, such as *Escherichia coli.* Carbonic anhydrases (CA, EC 4.2.1.1) in *E. coli* play a crucial role in bacterial metabolism and CO_2_/HCO_3_^−^ balance; therefore, they represent a suitable target for antimicrobial strategies. The nanoparticles were obtained using a green synthetic protocol that allowed conjugation of a natural fatty acid to hyaluronic acid (HA) under solvent-free conditions. Full characterization of the micellar aggregates was performed (diameter of the micelles, zeta potential, and drug release study). In vitro studies demonstrated that AZA loaded in HA-based nanoparticles significantly inhibited *E. coli* growth at concentrations as low as 0.5 µg/mL, whereas higher concentrations of free AZA were required, as previously reported. Additionally, encapsulated AZA disrupted glucose consumption in *E. coli*, indicating its profound impact on bacterial metabolism. These findings suggest that the HA–palmitate nanoparticle not only enhances the delivery and efficacy of AZA but also offers a strategy to affect bacterial metabolism.

## 1. Introduction

**Figure 1:**
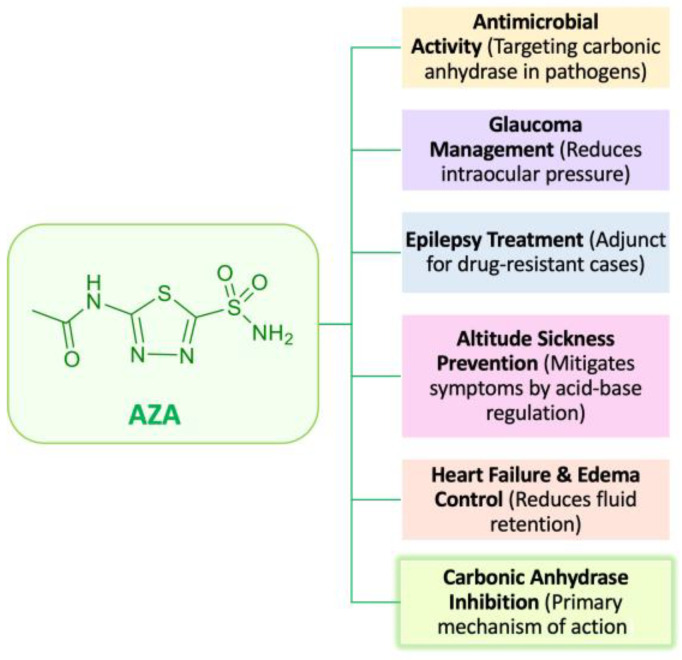
Therapeutic applications of AZA.

AZA was introduced into medical practice in the mid-20th century and has become a cornerstone in the treatment of various medical conditions [1]. Among the early researchers investigating its pharmacological properties, Henry D. Janowitz played a pivotal role in elucidating its effects in both animal models and human subjects [2,3,4]. By the 1950s, AZA was recognized for its potent inhibition of CA a key metalloenzyme that catalyzes the reversible hydration of carbon dioxide and regulates acid-base homeostasis [5,6,7,8]. AZA exerts broad physiological effects through the inhibition of human CA isoforms, making it a valuable therapeutic agent for multiple clinical applications [9,10,11]. This led to the approval of AZA by the United States Food and Drug Administration (FDA) for use in the management of many conditions [12,13,14]. For example, AZA has long been used to treat glaucoma by lowering intraocular pressure through reduced aqueous humor production [15]. Additionally, it serves as an adjunct therapy for certain forms of drug-resistant epilepsy [16], aids in managing congestive heart failure and edema by enhancing fluid excretion [17], and is effective in preventing and treating altitude sickness by inducing metabolic acidosis and stimulating ventilation [18]. In metabolic alkalosis, AZA helps to restore acid-base balance in critically ill patients [19]. More recently, AZA has been investigated for its potential antimicrobial effects, particularly against antibiotic-resistant pathogens [20,21]. Studies have shown that AZA derivatives can inhibit CAs in *Neisseria gonorrhoeae* and vancomycin-resistant enterococci (VRE), leading to bacterial growth inhibition [20,22,23]. Preclinical models have suggested that AZA-based compounds may be as effective as, or even surpass, linezolid in treating VRE [22,23,24,25]. These diverse pharmacological applications of AZA are summarized in Figure 1.

Despite its pharmacological versatility, concerns are associated with its poor solubility, poor bioavailability, and poor absorption in the body [26]. In addition, oral administration of AZA is often associated with systemic side effects, such as electrolyte imbalance and metabolic acidosis [27,28,29]. These limitations can be particularly detrimental to the application of AZA as an antimicrobial agent against Gram-negative bacteria, which are characterized by an outer membrane rather impermeable to drug penetration. While some nanotechnology-based methods are endowed with pharmacokinetic advantages for drug delivery, recent studies have also proven that in vivo cytotoxicity, oxidative stress, inflammation, and genotoxicity are often associated with the employed nanosystems [30,31]. Another important issue to be considered is the limited in vivo stability exhibited by several nanocarriers, since this strongly affects their safe circulation. In this regard, drug carriers based on natural polysaccharides can provide stable and biocompatible nanosystems for in vivo drug delivery. Among them, HA has been extensively investigated for biomedical application due to its non-toxicity, non-immunogenicity and biodegradability. In particular, amphiphilic HA conjugates, obtained by linking hydrophobic moieties, are able to self-assemble in nanoaggregates [32,33,34]. We have recently reported on amphiphilic HA derivatives obtained by chemical conjugation of natural fatty acids to the backbone of polysaccharides that are able to self-assemble in micellar aggregates and to efficiently encapsulate curcumin [35,36]. In this study, we report the development of an AZA-loaded HA-based nanocarrier that was fully characterized in terms of particle size and entrapment efficiency. Furthermore, the inhibitory effect of AZA encapsulated in the nanocarrier on the growth and metabolism of *E. coli* was investigated. *E. coli* was selected as a model organism, since a previous study proved that free AZA can impair bacterial survival by inhibiting its two CA classes, CynT2 (β-CA) and EcoCA*γ* (γ-CA) [37]. Compared with the effect of the free drug, AZA loaded in micellar HA-derivative aggregates significantly reduced the required dosage to achieve the desired effect, suggesting an enhanced efficacy in penetrating the bacterial membrane.

## 2. Results

### 2.1. Synthesis and Characterization of AZA Loaded HA–Palmitate Nanoparticles

**Figure 2:**
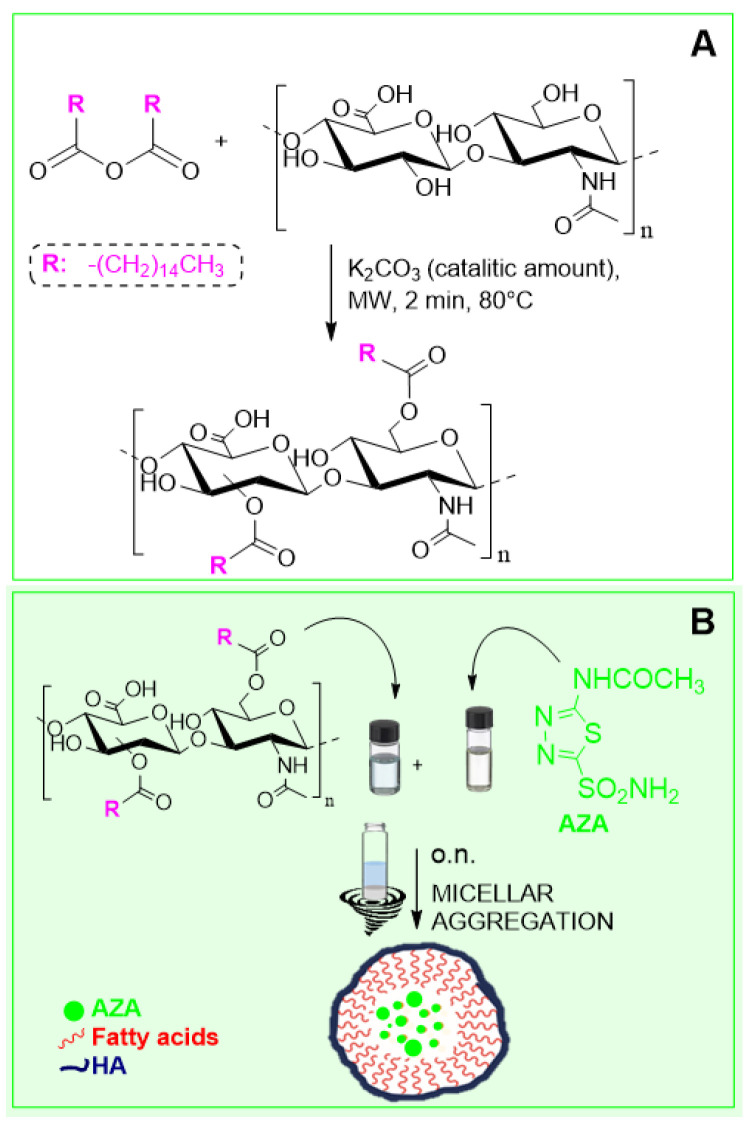
Synthetic strategy to HA–palmitate conjugates (**A**) and aggregation–encapsulation of AZA-loaded nanoparticles (**B**).

The HA–palmitic acid conjugate was prepared following a previously reported green chemical route [38]. This consists of a solvent-free procedure that executes a stepwise process in the same reaction vessel, where the activation of the palmitic acid carboxyl groups is followed by the subsequent esterification reaction with HA (Figure 2). Considering that CAC depends on the degree of functionalization of the hyaluronan with the fatty acid, we always employed a concentration of the polysaccharide conjugate above the average calculated value (~0.8 mg/mL) [39]. According to the previously reported protocol that analyzed HA conjugates’ properties as drug delivery nanosystem, the emulsion evaporation method was employed to form HA–palmitate aggregates that simultaneously encapsulate AZA.

**Figure 3:**
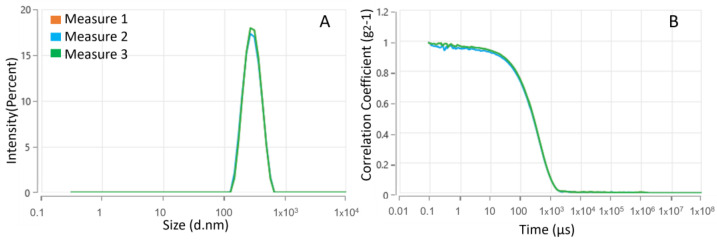
Size distribution (**A**) and intensity correlation functions of AZA–HA–palmitate nanoparticles (**B**).

AZA was dissolved in an organic solvent (chloroform), and HA–palmitate was dissolved in an aqueous solution of NaCl 0.9%. Subsequently, both solutions were mixed and left overnight under shaking at room temperature. Over time, the emulsion of the organic layer was broken, and the solvent evaporated. As a result, aggregates were formed and dispersed into the aqueous layer. Next, the size distribution was measured using dynamic light scattering (DLS), and the mean diameter of AZA-loaded HA–palmitate nanoparticles was found to be 265.9 nm ± 3.38 (Figure 3).

The polydispersity index (PDI) was 0.11 ± 0.011. Additionally, the ζ-potential was measured and found to be −20.54 ± 0.41 mV, as expected for the negative charge of the HA backbone carboxylic groups. To investigate AZA entrapment efficiency, the amount of drug encapsulated (final concentration) in HA–palmitate micelles was evaluated using UV spectroscopy. The disruption of the aggregates was performed in ethanol under sonication, and the free AZA was evaluated by measuring its absorbance at 264 nm. The best concentration for AZA embedded in the micellar delivery system was 5.12 µM.

**Figure 4:**
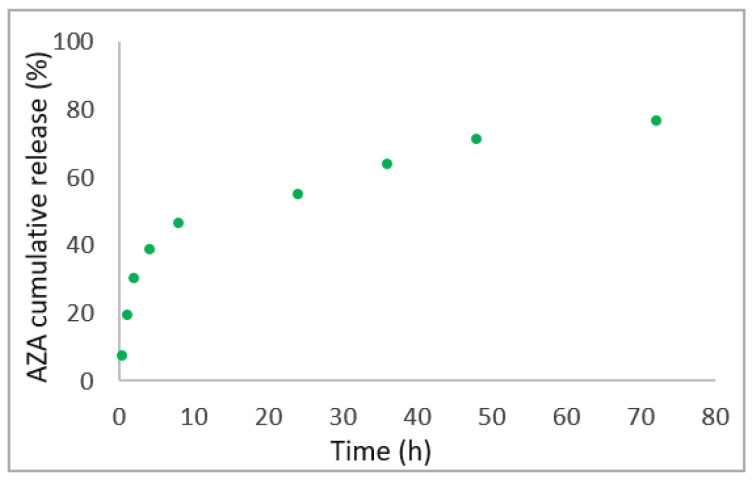
In vitro AZA release study. Green circles indicate the experimental data points collected at specific time intervals.

The drug release of the AZA from the HA–palmitate nanoparticles was evaluated over 72 h at room temperature in phosphate buffer (C = 0.1 M, pH = 7.4) on three different samples. A progressive drug release over time was observed until saturation was reached after 24 h. In Figure 4, the percentage of the drug found in the solution is a function of the time for a typical experiment. In particular, the drug released was estimated to be around 76.8% (SD = ±5.6) after 72 h [34,40,41].

### 2.2. Enhanced Efficacy of AZA–HA–Palmitate Nanoparticles

#### 2.2.1. Effect of AZA–HA–Palmitate Nanoparticles on *E. coli* Growth

**Figure 5:**
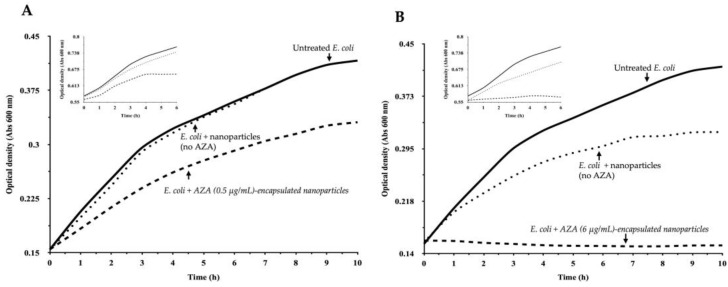
Effect of AZA–HA–palmitate nanoparticles on *E. coli* growth under different conditions. *E. coli* growth was monitored over time by measuring OD_600_ in MH medium under different treatment conditions. Two concentrations of AZA encapsulated in HA–palmitate nanoparticles were tested: 0.5 µg/mL (**A**) and 6 µg/mL (**B**). Each experiment was conducted under two conditions: Main graphs (**A**,**B**)—Nanoparticles were added at the time of bacterial inoculation (1.0 × 10^5^ CFU); Insets (**A**,**B**)—Nanoparticles were introduced during the exponential growth phase (OD_600_ = 0.5–0.6). Controls included untreated *E. coli* cultures (solid line) and cultures treated with empty nanoparticles without AZA (dotted line) at volumes of 50 µL and 150 µL. Growth curves were recorded hourly to assess the effect of AZA encapsulation on bacterial proliferation. Each data point represents the mean of at least three independent experiments. Graphs were analyzed using GraphPad Prism 6, and the data are presented as the mean ± standard deviation (SD).

To assess the effect of AZA encapsulated in HA–palmitate micelles on bacterial survival, we evaluated the growth of *E. coli* under different conditions. Bacterial growth was monitored hourly by measuring the optical density (OD) at 600 nm, which correlates with cell density. *E. coli* cells were incubated in MH medium with AZA–HA–palmitate at two concentrations of encapsulated AZA (0.5 µg/mL and 6 µg/mL). Aliquots of nanoparticles loaded with AZA at the desired concentrations were prepared by redispersing an appropriate amount of pre-synthesized AZA–HA–palmitate. The concentration of the micellar aggregates was consistently maintained at or above the estimated critical aggregation concentration (CAC) to ensure the stability of the drug delivery system. As controls, we included *E. coli* cultures without nanoparticles and those treated with empty HA–palmitate micelles. In this way, the potential effect on bacterial growth, arising from the presence of palmitic acid conjugated to HA, can be distinguished from the antimicrobial activity of AZA encapsulated in HA–palmitate micelles. Treatment with AZA loaded in nanoparticles resulted in a marked reduction in the AZA concentration required to achieve growth inhibition (Figure 5A,B).

Specifically, nanoparticles loaded with AZA were effective at concentrations as low as 31.2 µg/mL, which corresponds to the lowest concentration of free AZA that was previously tested [37]. Notably, at 6 µg/mL of AZA encapsulated in HA–palmitate nanoparticles, bacterial growth was completely inhibited (Figure 5B and its insert), representing an approximate 80% reduction in the concentration required to achieve full inhibition compared to free AZA. Each data point represents the mean of three independent experiments. This suggests that encapsulation in HA–palmitate micelles significantly enhances the antibacterial efficacy of AZA, allowing for potent growth inhibition at much lower concentrations compared to the free drug. The lower effective concentration of AZA embedded in nanoparticles could be attributed to improved interactions of the HA–palmitate micelles with the bacterial membrane.

#### 2.2.2. Impact of AZA-Loaded HA–Palmitate Nanoparticles on Glucose Consumption

**Figure 6:**
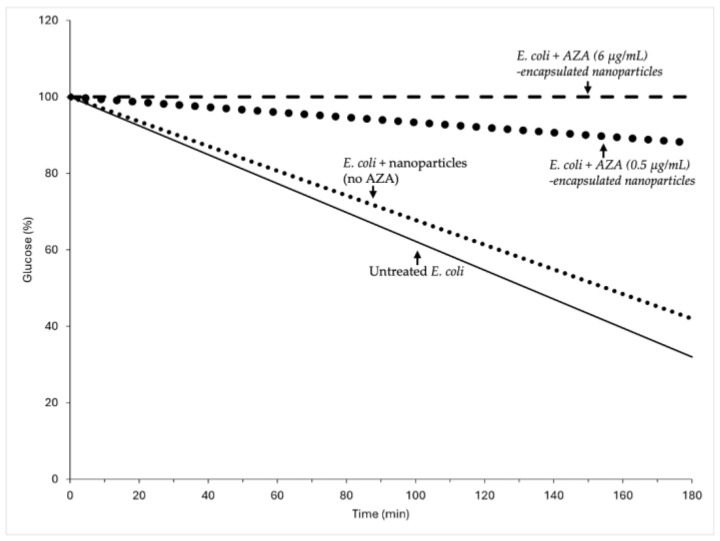
Effect of AZA-loaded HA–PA nanoparticles on glucose consumption by *E. coli*. Glucose consumption was monitored in *E. coli* cultures grown in a minimal medium supplemented with 0.4% glucose. Bacterial cells were treated with AZA-loaded HA–palmitic acid nanoparticles at two AZA concentrations (0.5 and 6 µg/mL), and glucose levels were measured every ten min for three hours. The control samples included untreated bacterial cultures (solid line) and cultures treated with empty nanoparticles without AZA (dotted line). The effects of AZA loaded in nanoparticles at 0.5 µg/mL and 6 µg/mL are shown by the large dots and dashed lines, respectively. A significant reduction in glucose consumption was observed upon treatment with AZA-loaded nanoparticles, with near-complete inhibition at the highest AZA concentration (6 µg/mL), suggesting enhanced metabolic disruption compared to the controls. Each data point represents the mean of at least three independent experiments. Graphs were analyzed using GraphPad Prism 6, and the data are presented as the mean ± standard deviation (SD).

Bacterial metabolism depends on efficient utilization of carbon sources, which play a critical role in biosynthetic pathways and energy production. Glucose, a key carbohydrate in microbial metabolism, is a reliable marker for assessing metabolic activity. In a previous study, free AZA reduced glucose consumption in a dose-dependent manner, with significant metabolic inhibition at concentrations above 31.2 µg/mL [39]. This reduction is likely due to the suppression of bacterial CAs, which are crucial to CO_2_/HCO_3_^−^ equilibrium and downstream metabolic processes. To determine whether the new formulation of AZA encapsulated in micelles enhanced AZA metabolic inhibition, we conducted experiments with AZA encapsulated in HA–palmitate nanoparticles at two concentrations (0.5 µg/mL and 6 µg/mL) (Figure 6).

AZA delivered by HA–palmitate nanoparticles significantly inhibited glucose consumption in *E. coli*, even at the lowest tested concentration of 0.5 µg/mL, where only 10% of the available glucose was consumed. At 6 µg/mL of AZA delivered by nanoparticles, glucose consumption was completely inhibited, with glucose levels remaining unchanged throughout the incubation period, indicating a clear suppression of bacterial metabolic activity. Each data point represents the mean of three independent experiments. In this case, AZA-loaded nanoparticles were added either at the time of bacterial inoculation or during the exponential growth phase. Despite these differences in timing, the results obtained from both conditions were similar, suggesting that the effect of AZA encapsulated in nanoparticles on bacterial metabolism is independent of when they are introduced. This indicates that AZA inhibition of bacterial CAs affects bacterial metabolism. This is plausible because CAs are involved in CO_2_/HCO_3_^−^ homeostasis, which is fundamental for bacterial metabolism. However, caution should be exercised against conflating metabolic activity with bacterial growth, as the two are often erroneously used interchangeably [42,43]. While metabolic activity reflects the rate of biochemical processes, growth measures the biomass increase over time. For example, during the stationary phase, cell numbers may continue to rise even as metabolism declines. Conversely, high metabolic activity can occur without a net increase in biomass, as in biofilms. Thus, assessing glucose consumption provides a more accurate snapshot of metabolic function than assessing growth alone [44,45,46]. Coupling both measurements allows for a clearer understanding of the bacterial physiology and the effects of AZA-loaded nanoparticles. Bacterial growth inhibition is dependent on the timing of AZA introduction, suggesting that AZA effects extend beyond metabolic inhibition. For example, during exponential growth, bacteria are more susceptible to disruptions in pH regulation, enzymatic activity, and secondary metabolic pathways, which could amplify the effect of AZA on replication and division. The new formulation of AZA encapsulated in micelles likely enhances its bioactivity by improving cellular uptake and prolonging its retention in bacterial cells, underscoring the potential of nanoparticles to enhance antibacterial efficacy.

## 3. Discussion

The *E. coli* genome encodes three CAs: β-, γ-, and ι-CAs. In our previous study, we heterologously overexpressed CynT2 (β-CA) and EcoCAγ (γ-CA) as His-tagged fusion proteins and achieved high-purity isolation from the cytoplasmic fraction using nickel-affinity chromatography [37]. The catalytic activity of the recombinant enzymes was confirmed by protonography, and CO_2_ hydratase activity was assessed using the stopped-flow technique. Kinetic constants were measured and compared to those of the human isoenzymes CAI and CAII. Both CynT2 and EcoCAγ proved to be highly efficient catalysts for CO_2_ hydration, with turnover number (k_cat_) values of 5.3 × 10^5^ s^−1^ and 5.7 × 10^5^ s^−1^, respectively. Additionally, they were effectively inhibited by AZA, a well-established pharmacological CA inhibitor, with inhibition constant (K_I_) values of 227 nM and 248 nM, respectively. Given the potent in vitro inhibitory effect of AZA on *E. coli* CAs, we previously investigated the impact of free AZA on bacterial cell growth and glucose consumption and demonstrated that free AZA interfered with *E. coli* growth and glucose consumption at concentrations higher than 31.2 µg/mL [37]. To further enhance drug efficacy and delivery, AZA was encapsulated in nanostructures obtained through the self-aggregation of an amphiphilic HA–fatty acid conjugate.

The results of this study provide compelling evidence for the enhanced efficacy of AZA loaded in HA–palmitate nanoparticles, particularly in targeting CAs in *E. coli*. In fact, the delivery of AZA embedded in nanosystems significantly improves cellular uptake, reducing the dosage required to inhibit bacterial growth compared with free AZA. Specifically, AZA loaded in nanoparticles was effective at concentrations as low as 31.2 µg/mL, the lowest concentration tested that still retained inhibitory activity, highlighting the increased potency conferred by the nanoparticle system. This observation is consistent with previous findings where nanoencapsulation of antimicrobial agents led to reduced minimal inhibitory concentrations (MICs) [47]. Similarly, polymeric nanoparticles carrying rifampicin exhibited enhanced efficacy against *Mycobacterium tuberculosis* due to improved bioavailability and intracellular accumulation [48].

The enhanced efficacy of the drug is likely due to more efficient penetration through the bacterial membrane. Although the HA–palmitate nanoparticles exhibited a net negative surface charge, which might typically reduce interactions with the similarly negatively charged bacterial membrane, this did not impair their antibacterial activity [49]. This suggests that the amphiphilic nature and structural features of the nanoparticle surface may compensate for the repulsive charge, promoting membrane interactions and facilitating uptake. Indeed, previous studies have shown that negatively charged lipid nanoparticles containing ascorbyl palmitate (a structural analog of palmitate) can maintain colloidal stability, interact favorably with biological membranes, and enhance delivery efficiency despite their surface charge [50]. These findings emphasize that surface chemistry, rather than net charge alone, plays a pivotal role in governing nanoparticle–cell interactions and delivery outcomes. Nanoparticle formulations are well-known to improve drug delivery and cellular uptake by promoting interactions with microbial membranes, often through increased permeability or membrane fusion [51]. For example, amphiphilic polymeric nanocarriers and liposomes have been shown to enhance antibiotic delivery via membrane destabilization and fusion, particularly in Gram-negative bacteria [32,33,52]. Similarly, cationic dendrimer-functionalized nanoparticles have proven improved penetration into bacterial cells by overcoming membrane barriers [53]. These studies support the notion that nanoparticle-based delivery systems, regardless of surface charge polarity, can substantially enhance intracellular delivery and bioavailability, supporting further mechanistic investigation. A hypothesized mechanism could be related to a more efficient interaction between the micellar delivery system and the impermeable outer membrane of Gram-negative bacteria. This is particularly relevant, considering that *E. coli* restricts the penetration of many therapeutic agents, including AZA. In addition, the biocompatible and biodegradable nanosystem can guarantee its implementation for in vivo circulation. Indeed, HA-based conjugates can be degraded to non-toxic fragments before renal excretion, since all the starting materials are provided by natural resources. Similar green synthetic approaches have been explored for other HA-based systems, where biocompatible polymers were conjugated under mild, catalyst-free conditions to ensure environmental safety and scalability [54]. In addition to the great advantage of using renewable resources as starting materials (HA and palmitic acid), it is worth emphasizing that an eco-friendly, cost-effective, and efficient protocol was employed to perform the conjugation reaction. A green procedure for developing drug delivery systems is widely encouraged, especially when considering the subsequent production of a scaled-up industrial process. The results indicated that AZA encapsulated in nanoparticles not only inhibited bacterial growth but also significantly reduced glucose consumption in *E. coli* cultures. The reduction in glucose uptake suggests disruption in bacterial metabolism, which is critical for energy production and biosynthetic pathways. This study highlights that AZA inhibition of bacterial CAs, which is crucial for maintaining CO_2_/HCO_3_^−^ equilibrium, plays a crucial role in this metabolic disruption. These findings imply that the encapsulated form of AZA may exert its effects beyond mere growth inhibition, potentially affecting various metabolic processes within bacteria [20,21,25]. The controlled release profile of AZA from HA–palmitate nanoparticles suggests that this formulation could provide prolonged therapeutic effects, reducing the need for frequent dosing compared with free AZA. This sustained release is advantageous in clinical settings as it may lead to improved patient compliance and reduced side effects associated with higher dosing frequencies. This study underscores the potential of nanoparticle delivery systems to enhance the bioactivity of existing antimicrobial agents, paving the way for novel therapeutic strategies against antibiotic-resistant pathogens.

## 4. Materials and Methods

### 4.1. Materials

HA sodium salt from *Streptococcus equi* (15–30 kDa molecular weight), palmitic acid, DIC, K_2_CO_3,_ CHCl_3_, AZA and Mueller–Hinton (MH) broth were purchased from Sigma-Aldrich (St. Louis, MO, USA). Spectrum™ Labs Spectra/Por™ 3 3.5 kD MWCO Standard RC Dry Dialysis Kits was purchased from Thermo Fisher Scientific (Waltham, MA, USA); *Escherichia coli* DH5α were purchased from Agilent Technologies (Santa Clara, CA, USA) The INNOVA 42 incubator was obtained from Eppendorf (Hamburg, Germany).

### 4.2. Synthesis of HA–Palmitate Conjugates

A total of 10 mg of palmitic acid and 0.5 equiv. of DIC were manually milled by using an agate mortar. The solid mixture, placed in a 0.5−2 mL microwave vial, was irradiated for 2 min at 80 °C in a microwave oven (Biotage Initiator+, Sweden AB, Uppsala, Sweden). In a subsequent step, HA and the palmitic anhydride were manually milled in the presence of 3 mg of K_2_CO_3_ (catalytic amount) to obtain the HA–palmitate derivative. The mixture, placed in a 0.5–2 mL microwave vial, was irradiated for 2 min at 80 °C in a microwave oven. After cooling at room temperature, the obtained solid sample was dissolved in aqueous 0.1 M HCl (~20 mL) in order to keep the pH at a value of 1–2. Then, it was placed in a 250 mL separatory funnel and was extracted with ethyl acetate in order to remove the unreacted palmitic acid. Subsequently, the aqueous layer was dialyzed (Spectra/por membrane cut-off (3.5 kD)) for 12 h in Milli-Q water. The final product was collected after the lyophilization process, yielding approximately 40%.

### 4.3. Preparation and Size Distribution Characterization of AZA-Loaded HA–Palmitate Micelles

AZA-loaded HA–palmitate nanoparticles were obtained by the evaporation method [55]. The following solutions were prepared: AZA was dissolved in chloroform, and a concentration of 50 µg/mL was reached (Sol. A); 3 mg of HA–palmitate was dissolved in 3 mL of 0.9% NaCl in water (Sol. B). The obtained solutions were combined (Sol. A + Sol. B), and the mixture was emulsified by stirring overnight at room temperature. Upon evaporation of the organic phase, the nanoparticles of HA–palmitate encapsulating AZA were dispersed in the aqueous layer. Thereafter, the aqueous dispersion (4.4 mL) was centrifuged at 13,000× *g* rpm for 10 min, and the insoluble free AZA was removed by performing size exclusion chromatography (Sephadex G50 column, GE Healthcare, Uppsala, Sweden). Then, the nanoparticles were characterized with Dynamic Light Scattering (DLS), employing ZETASIZER PRO (Malvern Panalytical, Malvern, UK; Almelo, The Netherlands). Other experimental settings were fixed as follows: temperature, 25.0 °C; cell, disposable micro cuvette (40–45 µL capacity).

### 4.4. Characterization of AZA Loaded HA–Palmitate Nanoparticles: Encapsulation Efficiency

The 4.4 mL solution of AZA-loaded HA–palmitate nanoparticles, eluted using size exclusion chromatography (SEC) [56], was employed to determine the encapsulation efficiency. Specifically, 400 µL of this solution were lyophilized and then reconstituted in 1.5 mL of ethanol (EtOH). The obtained mixture was sonicated for 30 min, and then the suspension was centrifuged at 13,000× *g* rpm for 7 min to eliminate the polysaccharide conjugates. Then, the amount of AZA encapsulated within the HA–palmitate nanoparticles was quantified using UV spectroscopy. The absorption spectrum of AZA showed a maximum at λ_max_ = 264 nm, with an extinction coefficient (ε) of approximately 12,435 L·cm^−^¹·mol^−^¹. The obtained measurement Abs(λ_264_) = 0.06358 allowed the calculation of a concentration, C= 5.12 µM. The total amount of AZA, evaluated in the volume of 4.0 mL, resulted in 5.01 µg. The UV spectra were recorded in the range of 220–350 nm by using 500 μL quartz cells and corrected for blank. Other experimental settings were fixed as follows: scan speed, 200 nm/min; data interval, 0.2 nm; response, 0.24 s; data interval, 0.2 nm; scan mode, continuous; bandwidth, 1.0 nm. The instrument used is the Jasco V-730 Spectrophotometer_ETCS-761.

AZA encapsulation efficiency resulted in a percentage of 10%. This value was calculated by the following equations [57]:

$$Encapsulation\;Efficiency\;\left(\%\right)=\frac{amount\;of\;loaded\;AZA\;in\;mg}{amount\;of\;AZA\;added\;in\;mg}\times 100.$$

### 4.5. In Vitro AZA Release Study

To investigate the drug release, AZA–HA–palmitate nanoparticles were dissolved in 2 mL of phosphate buffer (0.1 M; pH = 7.4) and placed in a dialysis bag (3.5 kD) that soaked in a beaker containing the same buffer solution. The dialysis was performed under magnetic stirring for 72 h. At predetermined time intervals, an aliquot (2 mL) of the dissolution medium was withdrawn, and its absorbance was measured at 264 nm. The same amount of fresh medium was added to the dialysis container to maintain the sink conditions. As 100% of encapsulated AZA gave an absorbance of 0.49, it allowed the calculation of the percentage of the released AZA over 72 h.

### 4.6. Bacterial Strains and Culture Conditions

Cells from the overnight culture were harvested by centrifugation at 4 °C (1500× *g*, 30 min), resuspended in fresh MH medium to a final optical density (OD) of 0.15 at 600 nm, and allowed to grow until OD_600_ reached 0.6. The pH of the medium was monitored to ensure optimal growth conditions. No antibiotics were added.

### 4.7. Treatment with AZA-Loaded HA-Based Nanocarrier

#### 4.7.1. Bacterial Growth Monitoring and Treatment Conditions

Experiments were conducted in 96-well clear TC-treated polystyrene microplates (Falcon, Männedorf, Switzerland) with a total volume of 200 μL per well. Cultures were incubated at 37 °C with continuous shaking at 80 rpm in an incubator integrated with a spectrophotometer plate reader (TECAN, Männedorf, Switzerland). The nanoparticle formulations used for treatment contained AZA at final concentrations of 0.5 μg/mL and 6 μg/mL. The following experimental conditions were applied: (a) Nanoparticles were added at the time of bacterial inoculation (1.0 × 10^5^ CFU, OD_600_ = 0.15); (b) Nanoparticles were introduced during the exponential growth phase (OD_600_ = 0.5–0.6). Control groups included untreated *E. coli* cultures and *E. coli* cultures treated with empty nanoparticles at volumes equivalent to those used for the 0.5 μg/mL and 6 μg/mL AZA treatments. Each experiment was performed in triplicate biological replicates, and OD_600_ was monitored at regular intervals using a SPARK multimode microplate reader (TECAN, Männedorf, Switzerland).

#### 4.7.2. Effect on Glucose Consumption

To evaluate the effect of AZA-loaded HA–palmitate nanoparticles on glucose metabolism in *E. coli*, the experiments described above were conducted using M9 minimal medium supplemented with 0.4% glucose. Metabolic inhibition was assessed by measuring glucose consumption using the phenol–sulfuric acid assay, as previously described [38]. An aliquot of the sample (5 µL) was used to analyze the sugar concentration. This was diluted to 200 mL with H_2_O and 1 mM EDTA and subjected to the phenol–sulfuric acid assay [38]. Briefly, 200 µL of 5% phenol and 1 mL of concentrated sulfuric acid were added to sample aliquots to reach a final reaction volume of 1.4 mL. The reaction mixture was allowed to stand for 15 min. The sample was then transferred to a cuvette, and absorbance was measured at 485 nm using a Varian Cary 50 Scan UV-Visible Spectrophotometer. Glucose consumption was calculated and compared to those observed in cultures treated with free AZA at concentrations of 0.5 μg/mL and 6 μg/mL. After 10 min of incubation for a total of six hours of exposure, glucose consumption was quantified and compared to those of the control groups. All experiments were performed in triplicate.

## 5. Conclusions

The encapsulation of AZA in HA–palmitate nanoparticles represents a promising approach to enhance the antibacterial efficacy of the drug against *E. coli*. The performed study demonstrates that this innovative formulation significantly reduces the concentration of AZA required to inhibit bacterial growth, achieving effective results at concentrations of drug as low as 0.5 µg/mL, compared to the higher concentrations needed for free AZA. This reduction in effective dosage not only suggests improved drug delivery but also indicates the potential to minimize the side effects associated with high amounts of AZA in circulation. Moreover, these findings highlight that targeting bacterial metabolism may be a strategy to overcome antibiotic resistance. Indeed, encapsulated AZA was shown to significantly disrupt glucose consumption in *E. coli*, indicating a profound impact on bacterial metabolism in addition to growth inhibition. Future research should focus on elucidating the mechanisms of action of AZA loaded in HA–palmitate nanoparticles. Additionally, the developed drug delivery system could be implemented for various bacterial infections, thus paving the way for new treatments that overcome antibiotic resistance.

## Data Availability

We will provide access to the data upon readers’ request.
